# PDX models reflect the proteome landscape of pediatric acute lymphoblastic leukemia but divert in select pathways

**DOI:** 10.1186/s13046-021-01835-8

**Published:** 2021-03-15

**Authors:** Anuli C. Uzozie, Enes K. Ergin, Nina Rolf, Janice Tsui, Amanda Lorentzian, Samuel S. H. Weng, Lorenz Nierves, Theodore G. Smith, C. James Lim, Christopher A. Maxwell, Gregor S. D. Reid, Philipp F. Lange

**Affiliations:** 1 Department of Pathology, University of British Columbia, Vancouver, BC Canada; 2 Michael Cuccione Childhood Cancer Research Program, BC Children’s Hospital Research Institute, 950 West 28th Avenue, Vancouver, BC V5Z 4H4 Canada; 3 Department of Pediatrics, University of British Columbia, Vancouver, BC Canada; 4 Department of Molecular Oncology, BC Cancer Research Centre, Vancouver, BC Canada

**Keywords:** Leukemia, Xenograft, Proteolysis, Proteome, N termini, Phosphorylation

## Abstract

**Background:**

Murine xenografts of pediatric leukemia accurately recapitulate genomic aberrations. How this translates to the functional capacity of cells remains unclear. Here, we studied global protein abundance, phosphorylation, and protein maturation by proteolytic processing in 11 pediatric B- and T- cell ALL patients and 19 corresponding xenografts.

**Methods:**

Xenograft models were generated for each pediatric patient leukemia. Mass spectrometry-based methods were used to investigate global protein abundance, protein phosphorylation, and limited proteolysis in paired patient and xenografted pediatric acute B- and T- cell lymphocytic leukemia, as well as in pediatric leukemia cell lines. Targeted next-generation sequencing was utilized to examine genetic abnormalities in patients and in corresponding xenografts. Bioinformatic and statistical analysis were performed to identify functional mechanisms associated with proteins and protein post-translational modifications.

**Results:**

Overall, we found xenograft proteomes to be most equivalent with their patient of origin. Protein level differences that stratified disease subtypes at diagnostic and relapse stages were largely recapitulated in xenografts. As expected, PDXs lacked multiple human leukocyte antigens and complement proteins. We found increased expression of cell cycle proteins indicating a high proliferative capacity of xenografted cells. Structural genomic changes and mutations were reflected at the protein level in patients. In contrast, the post-translational modification landscape was shaped by leukemia type and host and only to a limited degree by the patient of origin. Of 201 known pediatric oncogenic drivers and drug-targetable proteins, the KMT2 protein family showed consistently high variability between patient and corresponding xenografts. Comprehensive N terminomics revealed deregulated proteolytic processing in leukemic cells, in particular from caspase-driven cleavages found in patient cells.

**Conclusion:**

Genomic and host factors shape protein and post-translational modification landscapes differently. This study highlights select areas of diverging biology while confirming murine patient-derived xenografts as a generally accurate model system.

**Supplementary Information:**

The online version contains supplementary material available at 10.1186/s13046-021-01835-8.

## Background

Established clinical phenotypes and genomic alterations characterize pediatric acute lymphoblastic leukemia (ALL), the most common hematologic malignancy in early childhood [1–3]. These core genetic alterations, combined with other cooperating oncogenic drivers contribute to leukemogenesis.

The impact of animal models on leukemia research cannot be overstated [4]. Non-obese diabetic (NOD) severe combined immunodeficient mice (SCID) mice with deletion in interleukin 2 receptor gamma chain (IL2Rgamma), lack functional B- and T- cells, and have been proven to excellently engraft primary patient leukemia cells [4, 5]. Xenografts from B-cell ALL, T-ALL, and acute myeloid leukemia maintain patient-specific leukemogenic profile pertaining to the transcriptome, epigenome, and chromosomal aberrations [6–10].

The phenotype and functional capacity of a cell is largely driven by its proteins. Regulation of protein translation, degradation and post-translational modification leads to low correlation between the cellular proteome and genome / transcriptome [11, 12]. Thus, phenotypical and functional changes are frequently invisible at the genome level but apparent in the proteome. Following translation, proteins can be diversely modified, and made capable to simultaneously regulate multiple processes and pathways in separate cellular locations [13]. Formation of functionally different proteoforms [14] by post-translational modification (PTM) is frequent in diseases, including childhood cancers [15]. Protein phosphorylation and irreversible proteolytic processing are among the main PTMs that regulate cellular processes and contribute to the development and progression of cancer. The degree to which protein functional molecules and associated mechanisms broadly impact leukemic subtypes is yet to be understood. Such knowledge is crucial to determine if disease stratification based on proteome features can be attained, how well this complements known genomic subtypes, and what biological mechanisms provide stable molecules for precise therapeutic targeting. Functional and drug sensitivity studies in PDX models of leukemia can only be extrapolated to patients if the relevant pathways, protein networks and modifications are conserved between patient and murine model [16].

Total protein abundance, protein phosphorylation and limited proteolytic processing can be comprehensively studied using enrichment methods coupled to mass spectrometry-based proteomic techniques [14, 17]. Limited proteolytic processing is mediated by proteases/peptidases, and results in new stable protein fragments (proteoforms) with a distinct protein N terminus [18]. We recently developed a new enrichment procedure (High-efficiency Undecanal-based N Termini Enrichment, HUNTER) to extensively profile the N terminome of minimal disease samples [18, 19]. In addition to profiling proteolytic proteoforms, we and others have shown that the N terminome provides a reliable repertoire to verify the existence of truncated proteins, including stable fragments that are established biomarkers [20] and identify their functional relevance in biological processes such as apoptosis [19, 21–23].

A proteomic investigation of pediatric ALL prior and after transplantation in widely used mouse models will reveal proteome signatures characteristic of leukemic subtypes, and benchmark how effectively PDX models replicate the primary leukemic proteome. Importantly, characterizing global protein, limited proteolysis, and phosphoprotein patterns in pediatric ALL patients and corresponding PDX will identify select cellular pathways and processes for which protein and PTM abundance consistently differ between patient and matched PDX models.

## Materials and methods

### Experimental samples

#### Cell lines

B-ALL cell lines 380 (ACC 39), 697 (ACC 42) and T-ALL cell lines DND-41 (ACC 525), PEER (ACC 6) were purchased from DSMZ (Braunschweig, Germany). HeLa cells (American Type Culture Collection; cat. no. CCL-2) and ALL cell lines were cultured in RPMI-1640 media supplemented with 10% heat-inactivated fetal bovine serum (FBS) and 2 mM L-Glutamine (Gibco, Grand Island, NY) and maintained at 37 °C in 5% CO_2_. All selected cell lines met our set criteria of having pediatric origins, similar to the patient samples we studies (See Supplementary Methods).

#### Patient bone marrow and peripheral blood samples

Patient samples were collected with informed consent by Biobank staff during routine clinical care at BC Children’s Hospital (BCCH). All experiments were performed as approved by the University of British Columbia Children & Women’s Research Ethics, and conformed with standards defined in the WMA Department of Helsinki and the Department of Health and Human Services Belmont Report. Additional information on patient assessment and sample collection is detailed in Supplementary Methods.

Clinical cohort comprised of 8 patients with B-cell ALL (4=diagnosis, 4=relapse), 3 patients with T-cell ALL (2=diagnosis, 1=relapse), one patient with T-cell lymphoblastic lymphoma (T-LBL) and 2 patients with no detected leukemic blasts (normal or non-leukemia) (Table 1). Except for one T-ALL patient (T-01), diagnosis and relapse timepoints were not from the same patient. See Supplementary Methods for more description of sample cohort.

**Table 1:** Clinical information on Patients and corresponding NSG Xenografts

		Patient and disease characteristics at initial diagnosis					Disease characteristics of clinical samples (Dx and R) xenografted in NSG mice				Xenograft characteristics					
	Patient ID	Diagnosis	Sex	Age (years)	Cytogenetics	Risk group	Sample Time-Point	Time to Disease Progression (months)	Risk Group	Cytogenetics*	MNC Source	Patient Blast Content (%)	Injected viable MNCs	Xenograft ID	Time to Progression in PDX (days)	PDX Tumor Content (%)
1	B-01-Dx	B-ALL	F	11	Constitutional trisomy 21 Trisomy X	HR	Dx	NA	-	Constitutional trisomy 21 Trisomy X	BM	94	1M	B-01-Dx-PDX	146	92
2	B-02-Dx	B-ALL	F	3	Variant t(12;21) with ETV6/RUNX1 fusion Multiple chromosomal rearrangments [t(X;2), t(4;12;21), t(5;7), t(10;11), t(16;19)]	HR	Dx	NA	-	Variant t(12;21) with ETV6/RUNX1 fusion Multiple chromosomal rearrangments [t(X;2), t(4;12;21), t(5;7), t(10;11), t(16;19)]	BM	97	1M	B-02-Dx-PDXa	146	99
													1M	B-02-Dx-PDXb	130	98
3	B-03-Dx	B-ALL	F	2	Hemizygous deletion of p16 (CDKN2A) locus, del(9)	AR	Dx	NA	-	Hemizygous deletion of p16 (CDKN2A) locus, del(9)	BM	93	1M	B-03-Dx-PDXa	87	ND
													1M	B-03-Dx-PDXb	82	94
4	B-04-Dx	B-ALL	F	15	Cryptic t(12;21) with ETV6/RUNX1 fusion, Monosomy X, Complex abnormal karyotype, [(t,4;12)]	HR	Dx	NA	-	Cryptic t(12;21) with ETV6/RUNX1 fusion, Monosomy X, Complex abnormal karyotype, [(t,4;12)]	BM	95	1M	B-04-Dx-PDX	361	95
5	B-05-R1	B-ALL post B-cell Lymphoma	F	3	ND	-	R1	17	VHR	Normal: 46,XX	BM	68	1M	B-05-R1-PDX	255	98
6	B-06-R2	B-ALL	M	13	ND	-	R2	31	HR	Homozygous deletion of *CDKN2A* locus Complex karyotype [t8;9), t(13;17)]	BM	96	1M	B-06-R2-PDXa	246	96
													1M	B-06-R2-PDXb	240	98
7	B-06-R3			15	ND	-	R3	15	VHR	Homozygous deletion of *CDKN2A* locus Highly abnormal karyotype, [t(8;9), t(13;17)]	BM	90	1M	B-06-R3-PDXa	158	92
													1M	B-06-R3-PDXb	ND	ND
8	B-07-R3	B-ALL	M	15	NORMAL: 46,XY	-	R3	12	VHR	Homozygous deletion of *CDKN2A* locus	Blood	Numerous	1.6M	B-07-R3-PDXa	46	94
													1.6M	B-07-R3-PDXb	46	92
9	B-08-R2	B-ALL	F	17	Trisomy X	-	R2	24	VHR	Trisomy X RUNX1 amplification Complex karyotype, [t(2;12), t(3;8), t(4;17;14), t(12;15), t(12;16)]	BM	94	1M	B-08-R2-PDX	137	ND
10	T-01-Dx	T-ALL	M	5	Cryptic heterozygous deletion of CDKN2A	HR	Dx	NA		Cryptic heterozygous deletion of CDKN2A	BM	81	1M	T-01-Dx-PDX	69	92
11	T-01-R1	T-ALL		5		-	R1	60	HR	Cryptic heterozygous deletion of CDKN2A	BM	2	1M	T-01-R1-PDXa	61	98
		(Isolated CNS relapse with 2% bone marrow infiltration)											1M	T-01-R1-PDXb	55	ND
12	T-02-Dx	T-ALL	M	13	Monosomy 14 Interstitial deletion of chr. 6(q)	HR	Dx	NA	-	Monosomy 14 Interstitial deletion of chr. 6(q)	BM	90	0.7M	T-02-Dx-PDX	240	99
13	T-03-Dx	metastatic T-cell lymphoblastic lymphoma	F	10	Hyperdiploidy (+6, +8, +10, +11, +13, +19, +19)	ND	Dx	NA	-	Hyperdiploidy (+6, +8, +10, +11, +13, +19, +19)	BM	8	1M	T-03-Dx-PDX	208	94
14	NL-01	GATA2 mutation	M	6	Normal: 46, XY	NA	Non-leukemic	-	NA	-	BM	0	NA	NA	NA	NA
15	NL-02	Thrombocytopenia & macrocytosis	F	17	Normal: 46, XX	NA	Non-leukemic	-	NA	-	BM	0	NA	NA	NA	NA

*Abbreviations*: *ND* No data / Not determined, *NA* Not applicable, *M* Male, *F* Female, *CNS* central nervous system, *NSG* Non-obese diabetic scid gamma immunodeficient mouse, *MNC* mononuclear cell, *Dx* diagnosis, R relapse, R(1,2,3) = first, second, third relapse

Risk group = High Risk (HR), Average Risk (AR), Very High Risk (VHR)

Cytogenetics*: this is the same for diagnosis samples, but varies for relapse cases where the cytogenetics differed from that at diagnosis

#### Patient-derived xenografts

Primary ALL cells engraftment in mice was performed in accordance with an Institutional Animal Care and Use Committee-approved protocol (A15–0187). Viable mononuclear cells (MNCs) in sterile PBS were injected into the tail vein of 6- to 10- week old NOD.Cg-Prkdc^scid^/IL2rg^tm1Wjl^/SzJ (NSG) mice initially purchased from The Jackson Laboratory and bred and maintained in-house under pathogen-free conditions. Mice were euthanized at onset of overt leukemia which was determined by flowcytometry. See Supplementary Methods for more description of the criteria for determining overt leukemia, and selection of PDX for analysis. Tumor content in the spleen for PDXs used in our study were consistently greater than 90% (Table 1). For the 4 samples not evaluated for their tumor content, the animals had developed overt leukemia and highly enlarged spleens. All PDXs in this study were primary engraftments of patient leukemia.

#### Cell line-derived xenograft (CDX)

Pediatric ALL cell line 380 was injected via tail vein into NSG mice. Triplicate 380 cell samples were also prepared at the timepoint of injection and stored at − 80 °C for comparative analysis with 380 CDX. Mice were euthanized at onset of leukemia symptoms and spleen, bone marrow and liver samples were collected for analysis. Unlike PDXs, the primary leukemia-involved organ in 380 CDX was the liver, not the spleen, with human leukemia cells comprising 93% of harvested liver cells (compared to 16% in spleen).

Mononuclear cells isolated from harvested bone marrow, spleen and liver using standard procedure were viably frozen and stored.

### Ex vivo culture of primary and xenograft cells

Cryo-preserved pediatric ALL patient bone marrow (BM) and PDX cells were thawed at 37 °C and cultured in serum-free medium designed to support viable culture of hematopoietic cells. B-ALL was cultured in StemSpan™ SFEM II (StemCell, cat no. 09655) medium supplemented with 1X Stemspan™ CC100 (StemCell, cat no. 02690). T-ALL was cultured in StemSpan™ SFEM II (StemCell, cat no. 09655) medium supplemented with 100 ng/mL interleukin 2, and 25 μl/mL ImmunoCult™ Human CD3/CD28/CD2 T-cell activator (StemCell, cat no. 10970). Complete media was freshly prepared according to manufacturer’s instructions.

### Cell proliferation assay

Fifty thousand primary and PDXs cells were seeded per well in a 96-well plate and incubated at 5% CO2, 37 °C, for 24 h and 48 h. At 24 and 48 h timepoints, cells were labeled with the fluorescence-based CyQUANT Cell proliferation assay kit (Thermofisher, cat no. C35011). 2 μl of CyQuant dye was added to a total volume of 200 μl in each well. Cells were incubated for 1 h. Proliferating cells were distinguished using the ImageXpress Micro Confocal high-content imaging system (Molecular Devices, San Jose, CA) with the green fluorescent spectra (508/527 nm), and quantified using the MetaXpress high-content image acquisition and analysis software (version 5.2). Unpaired Student’s t-test was performed to compare proliferation rates in matched primary and xenograft cells.

### Immunoblot analysis

A total of 15–30 μg protein extracts were separated by 15% SDS-PAGE, transferred onto nitrocellulose membranes (Bio-Rad, Germany) and probed with antibodies against RB1 (Cell Signaling Technology, cat no. D20/9313), CDKN2A (p16 INK4A) (Cell Signalling Technology, cat no. D7C1 M/80772), and GAPDH (ProteinTech, Cat no. 6004–1-1 g). Primary antibodies were detected using goat anti-rabbit IgG (Thermo Fisher, cat no. SA5–35571) or goat anti-mouse IgG (Thermo Fisher, cat no. SA5–35521). Immunoblots were scanned using direct infrared fluorescence via the Odyssey system (LiCor Biosciences).

### Targeted next generation sequencing

Amplicon-based sequencing and variant determination was performed as described elsewhere [24], and is detailed in Supplementary Methods.

### Preparation of samples for mass spectrometry-based analysis

Except otherwise stated, reagents were purchased from Sigma Aldrich (St. Louis, Missouri, United States). Suspension cells cultured in T75 flasks were passaged up to five times. Biological triplicates were cultured separately. Cells harvested at greater than 90% confluency were washed twice with 1% PBS (Gibco, Grand Island, NY) and pelleted by centrifugation. Mononuclear cells isolated from patient bone marrow and blood, and from mice spleen, were quickly thawed, washed twice with 1X PBS, and harvested by gentle centrifugation. Cell viability performed on a 10 μl aliquot was above 70%.

Cell lines, patient and PDX samples were prepared for mass spectrometry-based analysis as detailed in Supplementary Methods.

### Liquid chromatography tandem mass spectrometry (LC-MSMS)

The methods for high pH fractionation and LC-MSMS are detailed in Supplementary Data.

At each stage of sample preparation and MS acquisition, − whole protein, phosphoprotein and N-termini, samples were randomized and batched accordingly.

### Statistical analysis and interpretation of MSMS data

DIA data were analyzed with Spectronaut Pulsar X (version 12.0.20491.3.15243, Jocelyn from Biognosys, Schlieren, Switzerland), as detailed in Supplementary Methods. PDXs in our cohort had spleen tumor content above 90%, and were therefore expected to have more human than mouse cells, and likewise human protein. To assess this, mass spectrometry data from PDX samples were searched against a combined protein fasta database consisting of human and mouse non-redundant proteins, and proteins were identified based on proteotypic sequences. At an FDR < 0.01%, protein identification was determined by the presence of at least one unique peptide. All PDX samples had less than 5% of murine proteins detected (Supplementary Fig. 1A). This points to the presence of limited mouse proteins in the xenograft samples studied. Following this, we proceeded to analyze patient and PDX data with only a non-redundant human fasta database.

Spectronaut output for 6397 identified proteins and related sample descriptions are provided in Additional files 1 and 2. A similar data for the N terminome experiment (6352 modified sequences) is in Additional files 3 and 4. Proteins, peptides, and N termini (acetylated and dimethylated sequences only), identified per sample in unfiltered Spectronaut output files are depicted in Supplementary Fig. 1B, C, and D respectively. Samples with lower blast count did not have markedly lower number of proteins and N termini in comparison to all other samples. Data for each experiment was further processed for additional statistical analysis as described in Supplementary Methods.

Precursor information from DIA analysis on phophopeptides were exported from Spectronaut and processed with the Peptide Collapse PlugIn (version v1.4.1) [25] in Perseus (version 1.6.2.2) [26] using settings described in Supplementary Methods. The number of phosphorylated sequences identified in each sample based on unfiltered Spectronaut output file is shown in Supplementary Fig. 1E. Following analysis with Peptide Collapse PlugIn, the resulting quantified data for 3531 phosphosites (Additional files 5 and 6) were used for further analysis.

The spectronaut output for 5939 proteins quantified in 380 cell line and cell line-derived xenograft is detailed in Additional file 7.

Quantified datasets were processed as explained in Supplementary Methods, and used for statistical analysis. Statistical analysis was done with two-tailed Student’s t-test. And where applicable, data was adjusted for multiple comparisons. The relevant multiple comparison test (Benjamini-Hochberg, False Discovery Rate, or Turkey’s multiple comparisons test) was applied to calculate adjusted *P* values, q < 0.05.

Data processing, and figure plotting was done in R, python, Perseus, GraphPad Prism and BioVinci. Hierarchical clustering analysis was performed with ‘Euclidean’ distance and ‘Average’ linkage on rows and columns. Equivalence tests were performed as detailed in Supplementary Methods.

Additional methods to characterize N-terminal peptides and to perform biological process and pathway enrichment are described in Supplementary Methods.

## Results

### Proteome differentiates pediatric B-ALL and T-ALL from non-leukemic cells

**Fig. 1:**
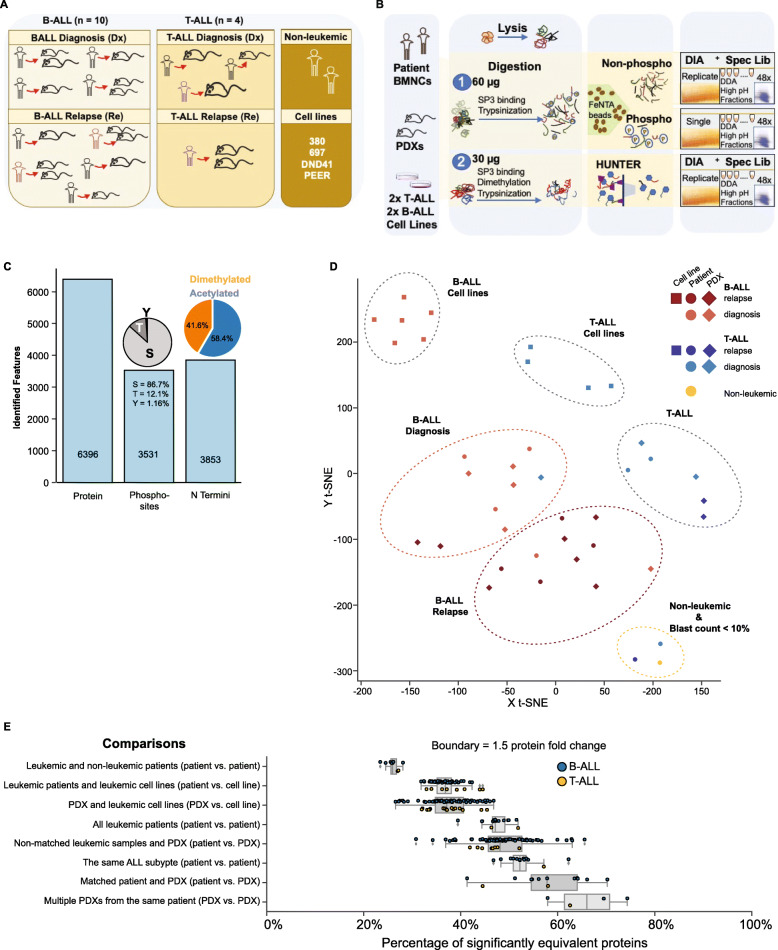
Proteomics stratifies pediatric ALL subtypes. **a** Composition of samples including number of childhood leukemia subtypes and disease stages, non-leukemic group, and leukemic cell lines analyzed. Same colours are maintained for ALL patient samples collected at different disease stages. Due to sample availability, our study did not include matched diagnosis and relapse samples from leukemic patients. **b** Detailed workflow for investigation of total protein, N Terminome, and phosphoproteome of pediatric ALL from minimal protein starting amount (60 μg protein for total and phosphoproteome study, 30 μg for N termini study). Proteome features were measured using Data Independent Acquisition (DIA) mass spectrometry methods, and analyzed with spectral libraries generated using combined information from DIA analysis and Data Dependent Analysis (DDA) of high pH fractionated sample pools. **c** Summary of quantified proteins (6396), phosphosites (3531), and N termini (3853) respectively. **d** T-distributed Stochastic Neighbor Embedding, t-SNE, plot following unsupervised analyses on average protein intensities (*N* = 5554) and K-means clustering on reduced dimensions from t-SNE. Clusters depict protein-level similarities and differences between model organisms (13 patients, 19 PDXs, 4 cell lines), disease subtypes (8 B-ALL, 3 T-cell leukemia), and disease stages (7 diagnosis, 6 relapse). **e** Each box plot shows the percentage of proteins with equivalent protein abundance (fold change < 1.5) for each combination of sample pairs compared within a sample group (See Methods for details of TOSTone test). The box plots show the percentage number of equivalent proteins from TOSTone tests performed between each ALL patient and the non-leukemic samples, leukemic patients and pediatric ALL cell lines, PDXs and pediatric ALL cell lines, all leukemic patients irrespective of disease subtype, non-matched patients and PDXs, patients with the same ALL subtype, matched patient and PDX pairs, and between multiple PDXs generated from a similar patient material. Box whiskers indicate standard deviation from the mean, and the mean equivalence is shown for each box plot. B-ALL and T-ALL samples for each comparison are represented with blue and yellow circles respectively

To test the hypothesis that murine xenografts of pediatric leukemia retain patient specific characteristics and to evaluate the extent to which the functional capacity of xenografted cells is altered by host-specific factors, we assembled a diverse cohort spanning multiple leukemia types, underlying genetic aberrations and disease stages (Table 1). Mononuclear cells from bone marrow aspirates of 13 pediatric ALL patients were each transplanted in NOD/SCID/IL2 gamma-receptor null (NSG) mice. To evaluate the reproducibility of xenografts, two mice were engrafted when sufficient primary patient cells were available. Overall, thirteen primary leukemia samples, 19 corresponding xenograft leukemia, 2 non-leukemic samples, and 4 pediatric leukemia cell lines (B-ALL: 697, 380; T-ALL: DND41, PEER) were analyzed using multiple mass spectrometry-based proteomics strategies (Fig. 1a and b) and targeted next generation sequencing. To control for the effect of blast count in patient bone marrow cell population, two samples originating from ALL patients with less than 10% leukemic blasts composition in the bone marrow compartment (higher proportion of normal hematopoietic mononuclear cells) were included in the study (Fig. 1a and Table 1). The number of blood and bone marrow mononuclear cells attainable from pediatric patients is limited. For this study we had access to 0.6–5 million primary cells per patient. Protein yield per sample ranged from 70 to 281 μg. As a first step of normalization, a defined protein amount was used for proteomics studies on all samples. Starting from 60 μg protein per sample, in crude cell lysate, we identified in total, 6396 proteins (Fig. 1c), and 3531 phosphosites (Fig. 1c). Likewise, 3853 N termini were identified from 30 μg starting protein amounts (Fig. 1c). We assessed the precision of our quantification by determining the coefficient of variation (CV) between repeat injections. Protein-level and N termini average CV were below 6 and 21% respectively (Supplementary Fig. S1F and G). Our methods therefore support robust and sensitive investigation of the proteome of childhood acute lymphoblastic leukemia.

Unlike the homogenous blast population in leukemia, normal bone marrow consists of a complex mixture of cell types. In our patient cohort, increased bone marrow cellularity and high blast cell population correlated with a loss of mature myeloid, erythroid, and lymphoid cell populations (Table 2). An overlap of proteins represented in non-leukemic and diseased categories (Supplementary Fig. 2A and B) revealed a large protein subset common to patients and PDX was not detected in non-leukemic samples. The proteome landscape of pediatric B- and T-ALL patients is clearly different from non-leukemic samples and reflects pathological features of leukemic bone marrow before xenotransplantation.

**Table 2:** Bone marrow morphology for clinical cohort

Patient characteristics		Sample features			Bone marrow differential (per 100 cells counted)																		
					Myeloid								Erythroid				Lymphoid						
Leukemia Subtype	Sample ID	Total Cellularity	Abnormal Infiltrate of immature cells	Megakaryocytes	Pro myelocyte	Myelocyte	Meta myelocyte	Band cells	Neutrophils	Eosinophils	Basophils	Monocyte	Pro Normoblast	Early Normoblast	Intermediate Normoblast	Late Normoblast	Blast cells	Pro lymphocyte	Lymphocyte	B lymphocyte	T lymphocyte	Natural Killer Cells	Plasma cells
B-ALL	B-01-Dx	Increased >95%	Present	Rare	-	-	-	-	-		-	-	-	-	2	-	94	-	4	-	-	-	-
	B-02-Dx	Increased	Present	Decreased	-	-	-	-	-	-	-	1	-	-	-	-	97	-	2	-	-	-	-
	B-03-Dx	Increased (100%)	Present	Present	-	-	1	-	-	-	-	1	-	-	-	-	93	-	5	-	-	-	-
	B-04-Dx	Increased (>95%)	Present	Present	-	-	1	-	-	-	-	-	-	-	1	-	95	-	3	-	-	-	-
	B-05-R1	Increased	Present (80%)	Present	-	1	4	2	2	2	-	4	-	4	12	-	68	-	1	-	-	-	-
	B-06-R2	Increased >95%	Present	Reduced	-	-	-	-	-	-	-	-	-	-	1	-	96	-	3	-	-	-	-
	B-06-R3	Increased >95%	Present	Present	-	2	-	-	-	-	-	-	-	1	3	-	90	-	4	-	-	-	-
	B-07-R3	ND	ND	ND	-	-	-	-	2.31	0.77	-	0.77	-	-	-	-	-	-	6.93	-	-	-	-
	B-08-R2	Increased (100%)	Present	Rare	-	-	-	-	1	-	-	-	-	-	-	rare	94	5	-	-	-	-	-
T-ALL	T-01-Dx	Increased >95%	Present	Present	-	-	-	1	1	1	-	-	-	1	2	-	81	-	13	-	-	-	-
	T-01-R1	Increasesd (95%)	Absent	Reduced	2	4	5	6	27	-	-	1	1	2	4	40	2	-	6	-	-	-	-
	T-02-Dx		Present	Reduced	-	-	-	2	3	-	-	-	-	-	1	2	90	-	2	-	-	-	-
T-LBL*	T-03-Dx	Low, normal (50 - 60%)	Present	Normal	3	6	8	10	8	6	-	1	1	5	21	4	8	-	16	-	-	-	3
Non-leukemic	NL-01	Low, normal (50-60%)	None	Adequate	-	10	18	9	18	2	-	2	-	-	16	-	0	-	25	-	-	-	-
	NL-02	Reduced	None	Reduced, normal	1	8	3	2	7	2	1	2	1	6	18	9	3	-	36	-	-	-	1

*Dx* diagnosis, *R* Relapse, * = T-cell lymphoblastic lymphoma (T-LBL)

### The proteome of xenograft ALL closely resemble matched patient proteome

Unsupervised T-distributed Stochastic Neighbor Embedding (t-SNE) dimensionality reduction followed by K-means clustering on protein abundance measurements categorized samples in distinct groups: non-leukemic, B-ALL at diagnosis and relapse, T-ALL cases, and leukemic cell lines (Fig. 1d). Xenografted cells consistently clustered alongside their primary counterparts. As was anticipated for specimen consisting primarily of normal mononuclear cells, samples from the two patients with minimal disease burden (T-01-R1 and T-03-Dx, blast count < 10%) consistently associated more with the non-malignant specimen (Fig. 1d). Mice that received blasts from these patients developed overt leukemia, and their protein profile associated more closely with xenograft leukemic samples than with their primary samples. The average Spearman’s correlation scores for protein abundance in T-ALL patient and corresponding xenograft mice was 0.83 (SD=0.06) (Supplementary Fig. S3A), with lower correlations (average=0.67, SD=0.06) observed between primary leukemias with lower disease burden and their matched PDXs. B-cell leukemia samples had average correlation score of 0.88 (SD=0.02) (Supplementary Fig. S3B). Proteins involved in known signaling pathways affected in pediatric leukemia also correlated strongly between primary and PDX leukemias (Supplementary Fig. S3A and B).

We next tested for formal equivalence of protein abundance. We defined a protein as equivalently expressed between any two samples when the abundance differs significantly less than 1.5-fold. On average 26% of proteins were equivalent between leukemic and non-leukemic patients (Fig. 1e). In contrast, 53% of proteins were equivalent between patients from the same ALL subtype, and this reduced to 47% between all patients irrespective of disease type. Matched patient and PDX pairs had on average 57% equivalence of protein abundance. Multiple PDXs from the same patient retained the highest protein equivalence (mean = 66%). A low protein abundance equivalence was found between patients and pediatric ALL cell lines (37%) as well as between PDXs and cell lines (37%). These results show that patients and their corresponding PDXs have a high level of similarity at the protein level, and PDXs from the same patient are alike at the protein level.

**Fig. 2:**
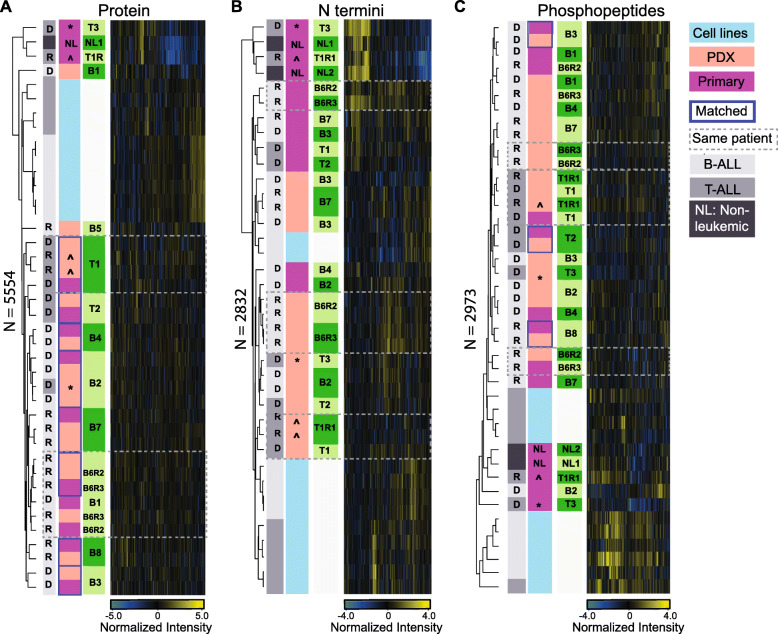
Pediatric ALL proteome landscape in patients, PDXs and leukemic cell lines. **a**, **b**, **c** Hierarchical clustering map following unsupervised analyses on (A) 5554 quantified proteins, (B) 2832 quantified N termini, and (C) 2973 quantified phosphopeptides. Values plotted in heat maps are z-score normalized. Paired patient and xenograft samples are highlighted with blue border lines. The symbols ^ and * represent two samples, T-01-R1 and T-03-Dx, with low disease involvement of the bone marrow (< 10% of blasts), and their corresponding PDXs

Unlike protein level clustering (Fig. 2a), unsupervised hierarchical clustering of protein N termini abundance grouped samples primarily by host and leukemia type (Fig. 2b). Although matched patient and PDX did not co-cluster, a positive correlation (B-ALL average=0.62, SD=0.06; T-ALL average=0.60, SD=0.03) was retained between individual patients and their corresponding xenografts (Supplementary Fig. S4). Of note, the N terminome of patients with low leukemic burden had little or no correlation with their PDX recipients (average correlation=0.13, SD=0.04). Also, the N terminome of non-leukemic patients correlated positively with that of patients with low leukemic burden (Supplementary Figs. S4B and D).

From minimal clinical material (60 μg crude protein lysate), we identified 3513 class I phosphosites (localization probability > 0.75) from 3378 phosphorylated sequences, corresponding to 1450 phosphoproteins. Unsupervised hierarchical clustering on 2973 quantified phosphosites showed no consistent association between matched primary and PDX leukemias (Fig. 2c). Limited availability of clinical material made replica analyses impossible for N termini and phosphorylation experiments preventing more in-depth statistical analyses like equivalence testing.

Compared to pediatric ALL cell lines and non-leukemic bone marrow cells, the proteomes of xenograft ALL most closely resemble that of their patient at protein level, while at PTM level model- and disease-specific similarities exceed paired patient-PDX relationships. Subsequent analysis focused on patient and corresponding PDXs and excluded cell lines.

### Relapse specific changes in patients are retained in PDXs

**Fig. 3:**
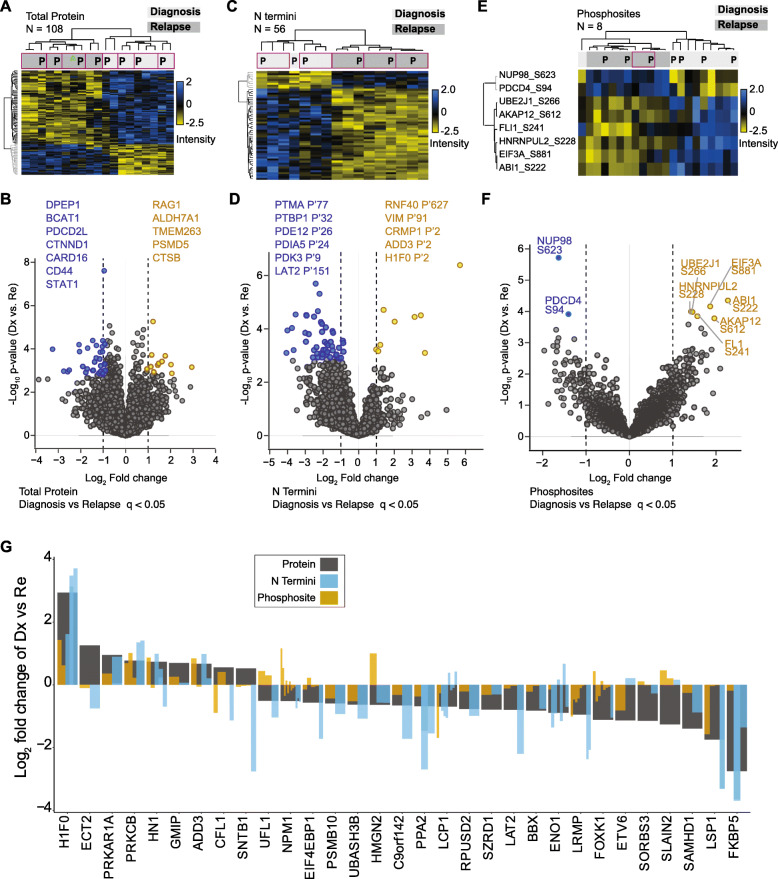
PDXs reflect proteome changes that distinguish diagnosis and relapse conditions in patients. **a** Heatmap on z-score normalized intensities of 108 proteins that significantly distinguished diagnosis from relapse B-ALL conditions (supervised hierarchical clustering, unpaired Student’s t-test, FDR < 0.05). Purple border highlights paired patient (P) and PDX samples. **b** Fold change and *p*-value profiles of the top differentiating proteins (FDR < 0.05) in diagnosis and relapse samples. **c** Supervised hierarchical clustering on 56 N termini that significantly distinguished (unpaired Student’s t-test, FDR < 0.05) diagnosis from relapse samples. Plotted data was z-score normalized. Purple border highlights paired patient (P) and PDX samples. **d** N termini with significant differences between diagnosis and relapse time-points (FDR < 0.05) are highlighted in a scatter plot. **e** Supervised hierarchical clustering on 8 phosphosites with differential abundance in diagnosis and relapse cases (ANOVA test, FDR < 0.05, z-score normalized values plotted). Purple border highlights paired patient (P) and PDX samples. **f** Scatterplot shows significantly dysregulated phoshosites in diagnosis and relapse patients (q value < 0.05). **g** Fold change (Log_2_) of 31 proteins differentially regulated proteins (grey bars) in diagnosis compared to relapse samples that also had phosphosites and N termini quantified. Log_2_ fold change (diagnosis vs. relapse) of associated protein phosphosites (brown bars) and N termini (blue bars) are inserted within the protein bar plots

Our proteomic profiles could distinguish pediatric B-ALL and T-ALL subtypes at the protein (Fig. 1c), phosphoprotein, and N termini level (Fig. 2). Focusing on B-ALL where we had more matched patient and PDX samples, we investigated if we could detect specific changes between diagnostic and relapse disease timepoints (Fig. 1a) and if these differences were retained in xenograft leukemia. The profile of 108 proteins with significant abundance changes (unpaired two-tailed Student’s t-test, FDR< 0.05) between B-ALL diagnostic and relapse patients was highly correlated between patient and matched xenograft (Fig. 3a, Supplementary Table S1). This included known proteins previously associated with leukemogenesis, such as CD44 and STAT1 (Fig. 3b), as well as multiple uncharacterized markers. Differences at the N termini level were also largely comparable in patients and paired PDXs for 56 N termini that distinguished diagnosis and relapse conditions (unpaired two-tailed Student’s t-test, FDR< 0.05) (Fig. 3c). Neo N termini (See Methods for definition) from leukemia-associated proteins such as PDK3 and LAT2 (Fig. 3d) were elevated in relapse cases. Relapse associated changes we detected at the phosphosite levels were recapitulated in xenografts but showed lower conservation between matched pairs (FDR< 0.05) (Fig. 3e and f). We determined the overlap between N termini, phosphosites, and proteins in diagnosis compared to relapse samples. At a protein-level threshold of *p* < 0.05 and fold change greater than or equal to ±0.5, 130 had matching N termini (Fig. S5A), 96 had matching phosphorylated sites (Fig. S5B) and 31 proteins had corresponding N termini and phosphosite features (Fig. 3g). The majority of PTM features follow the pattern of protein dysregulation. Overall, the proteome and PTM profiles that distinguish diagnostic from relapsed childhood ALL are predominantly recapitulated in PDXs.

### Proliferation and immune response processes differ between PDXs and patients

We next investigated if the minor differences between patients and matched xenografts affect random proteins or if they represent specific cellular processes and signaling pathways. These differences were determined by analyzing uniquely expressed proteins, differentially expressed proteins, and enriched protein complexes.

We first compared biological processes and pathways enriched in proteins that were uniquely identified in patients or in PDXs (Supplementary Fig. S6A and B). Multiple immune-related and metabolic pathways were significantly enriched in both B- and T-ALL patients when compared with xenografts (Supplementary Table S2, Supplementary Figs. S6C and S7A). In PDXs, processes linked to cell division and regulation of cyclin-dependent protein serine/threonine kinase activity were enhanced (Supplementary Fig. S7B).

**Fig. 4:**
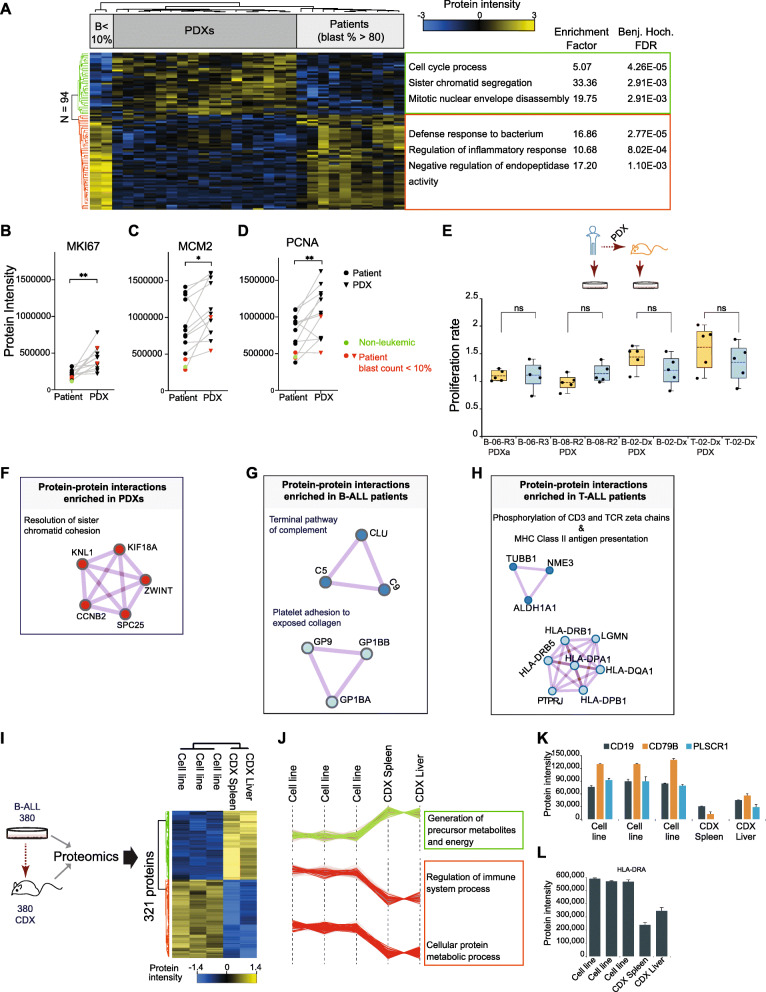
PDXs and CDXs demonstrate differences in cell proliferation and immune response processes compared to patients. **a** Supervised hierarchical clustering of differentially regulated proteins (Two-tailed Student’s t-test, FDR < 0.05) in all patients compared to all PDXs. Heatmap depicts z-score normalized intensities for 94 proteins. Processes and pathways enriched in proteins in each of two distinct clusters are displayed (Fisher exact test, Benjamini-Hochberg FDR < 0.02). Enrichment test was performed using annotations for 5554 total proteins as background. Patient samples with reduced bone marrow disease involvement (blast count, B < 10%) are indicated. **b-d** Two-tailed paired Student’s t-test on (B) MKI67, (C) MCM2 and (D) PCNA protein abundance in 12 matched ALL patient and PDX pairs. Samples from the non-leukemic patient and from the two patients with blast infiltration less than 10% are highlighted in green and red respectively. Symbols * and ** denote *p*-values of 0.001 (MKI67), 0.03 (MCM2), and 0.006 (PCNA). **e** Proliferation rate of five matched primary and xenograft cells expanded in an ex vivo culture system. Experiment was performed in quintuplicates. **f**, **g**, **h** Proteins unique to either PDX or host, and found to be involved in established interactions and functional roles in (F) both B-ALL and T-ALL xenografts, (G) B-ALL patients, and (H) T-ALL patients (See methods for description of analysis in Metascape). **i** Schema for proteomics analysis on 380 cell line and 380 cell line-derived xenograft (CDX). Supervised hierarchical clustering of 321 proteins differentially regulated in cell lines (biological triplicates) and in liver and spleen from 380 CDX. Statistical analysis was performed with unpaired Student’s t-test, FDR < 0.05. **j** Biological processes enriched in proteins markedly abundant in 380 CDX (green cluster) and processes deficient in 380 CDX (red cluster). **k**, **l** The abundance of protein receptors involved in immune regulation is markedly different in 380 cell line and CDX

Next, we identified processes enriched in proteins showing altered protein abundance between patient and PDXs. The 94 proteins with altered abundance (two-sample Student’s t-test, FDR< 0.05) between patients and xenografts clustered in two main groups (Fig. 4a, Supplementary Table S3). A Fisher exact test to determine non-random Gene Ontology (GO) associations between these proteins and all proteins quantified revealed that proteins with lower abundance or absence in PDX specifically associated with immune and inflammatory cellular response. This included proteins S100A12, S100A9, CHI3L and S100A8, with major roles in cytokine promotion and chemokine production. In contrast, proteins related to cell cycle and mitosis were enriched in PDXs when compared to patients. These results concurred with enriched patterns attributed to proteins detected or missing between PDXs and murine hosts. To confirm if cell proliferation was indeed increased in xenografts when compared to patients, we quantified the abundance of known proliferation markers across all paired samples (Figs. 4b-d, Supplementary Table S4). MKI67, PCNA and MCM2 levels were significantly increased in all PDXs compared to their primary counterparts (paired Student’s t-test, *p*< 0.05). PCNA and MCM2 levels had a downward trend in xenograft leukemia from sample B-01-Dx.

To determine if the observed differences in cell proliferation could be largely due to the different cell states in patient bone marrow compared to xenograft spleen, we measured cell proliferation in paired patient and xenograft samples ex vivo (Fig. 4e). We hypothesized that such an assay will point to the basal proliferative capacity of patient and xenograft cells in a defined culture system. Our results show that for five matched primary and xenograft samples, expanded cells and patient blasts have the same proliferative capacity when exposed to the same environment (Fig. 4e). In line with gene expression studies [7], these data suggests that the PDX environment strongly contributes to the enhanced cell cycle, mitosis, and proliferation signatures in xenografts.

Protein-protein interaction enrichment analysis (See Methods) further identified pathways containing protein complexes that might be disrupted or functionally compromised by altered expression of complex members in either patients or PDXs. For ALL xenografts, a set of 5 enriched interacting proteins were functionally assigned to the pathway “Resolution of Sister Chromatid Cohesion”, a key step in mitotic anaphase during cell division (Fig. 4a). In B-ALL, components of two pathways were identified. Proteins CLU, C9, and C5, were functionally linked to the “Terminal pathway of complement”. Also, GP9 (CD42a), GP1BA (CD42b), and GP1BB(CD42c) activities were associated with the functional pathway “Platelet adhesion to exposed collagen” (Fig. 4f). In T-ALL “Phosphorylation of CD3 and TCR zeta chains, and MHC Class II antigen presentation were the most enriched functional pathways represented by interacting proteins (Fig. 4g). The absence of these proteins could be linked to the lack of a human immune system in NSG mice. To further examine if the differences in immune and cytokine signatures reflect deficiencies in xenograft or patient cell state, we generated pediatric cell line-derived xenografts (CDXs, see Methods). Similar to PDXs, B- and T-ALL cell lines lacked or minimally expressed proteins including serpins and S100 proteins, that regulate immune and inflammatory response systems in patients (Supplementary Fig. S8A). Mass spectrometry-based proteomic analysis of the B-ALL 380 cultured cell line, as well as spleen and liver from 380 CDXs confirmed the absence of these proteins in CDXs (Fig. 4i and j). Leukemia in 380 CDX localized to the liver (93% human cells) and not spleen (16% human cells), and CDX liver best reflected the proteome of B-ALL 380 in comparison to xenograft spleen (Supplementary Fig. S8B). In addition to this, 380 CDX samples had significantly reduced levels (unpaired two-tailed Student’s t-test, FDR< 0.05) of protein receptors involved in immune regulation (Fig. 4k and l). When compared with 380 cell lines, 380 CDX spleen and liver had higher amounts of MCM2 proliferation marker, while PCNA showed an increased but not significant trend (Supplementary Fig. S8C).

As expected, the immune deficiency of the murine host was accompanied with absence of specific proteins and protein complexes from the xenografted cells, validating our approach of identifying functional differences. Xenografted cells showed enrichment in cell cycle pathways which could be validated further by overexpression of known proliferation markers, indicating an increased proliferative potential of xenografts.

### Xenografts recapitulate proteome response to structural genomic changes in patients

**Fig. 5:**
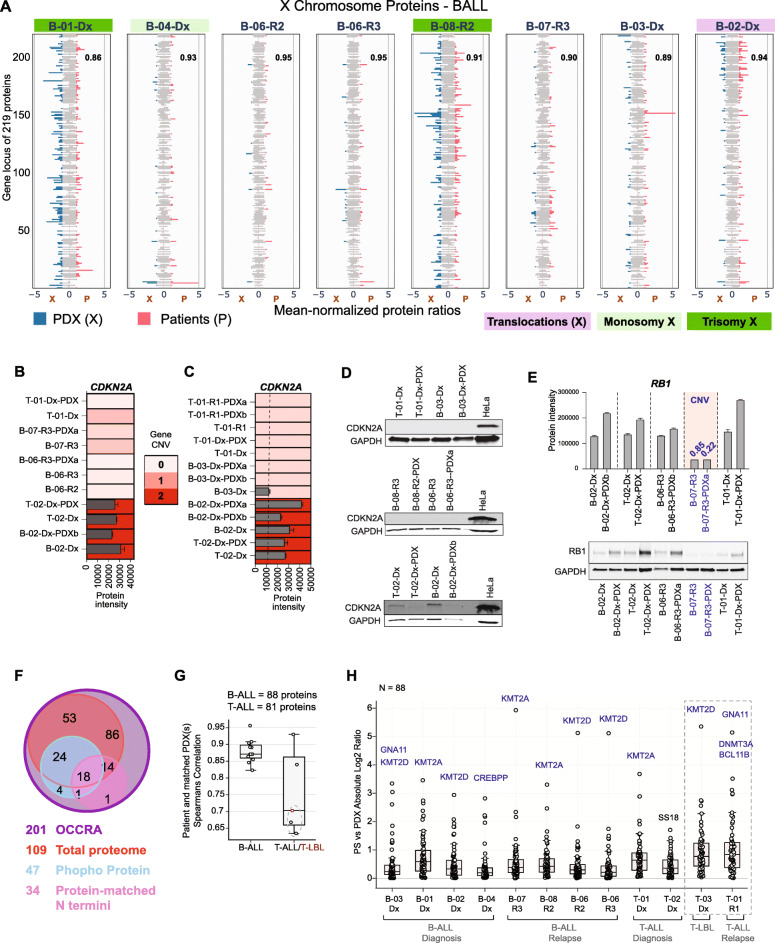
Pediatric ALL proteome reflects consequences of genomic changes. **a** Mean-normalized protein intensities of 219 quantified protein products from genes on chromosome X in 8 patients (P, pink bars) and in their matched PDXs (X, blue bars). Average intensities are reported for multiple PDXs engrafted with the same patient leukemia. Patients with structural defects in their X chromosome are highlighted – translocation in purple, monosomy X in light green, and trisomy X in dark green. Pearson correlation coefficient score for each matched patient and PDX pair is provided inset. **b** *CDKN2A* copy number determined by targeted gene sequencing in matched patient and PDXs, and CDKN2A protein level in same patient and xenograft samples. **c** CDKN2A protein level in all samples with one or two copies of *CDKN2A*, as confirmed by clinical cytogenetics and/or targeted gene sequencing. **d** Immunoblot analysis of primary and PDX samples with no copy of *CDKN2A* (B-06); one copy of *CDKN2A* (T01 and B-03), and two copies of *CDKN2A* respectively. A HeLa positive control is included in each panel. Due to limited availability, different sample amounts were loaded on the same gel and is apparent in the lower GAPDH control levels for two PDX samples. **e** *RB1* copy number variation determined by targeted gene sequencing, and RB1 protein level measured by mass spectrometry and immunoblot in same paired patient and xenograft samples. **b**, **c**, **e** Bar plots are mean protein abundance from two DIA technical replicates, and error bars represent the standard deviation from the mean. CNV = copy number variation, and is highlighted in blue. **f** Summary of 201 OCCRA panel genes profiled in total protein (109), phosphoprotein (47), and matched protein N termini (34) data from clinical samples. **g** Spearman correlation scores for quantified OCCRA proteins in paired patient and PDX leukemia samples (B-ALL = 88 proteins, T-ALL = 81 proteins). Points highlighted with dashed semi-circles represent values from comparing protein abundance in patients with low blast count (< 10%) and their matched PDXs: T-01-R1 and T-01-R1-PDXa, T-01-R1 and T-01-R1-PDXb, T-03-Dx and T-03-Dx-PDX respectively. **h** Absolute Log_2_ ratios of protein intensities (*N* = 88) in 12 ALL patients and their corresponding PDX. Proteins with highest variation between both models are highlighted in blue. Values form patients with reduced bone marrow involvement (< 10% blasts) and their corresponding PDXs are highlighted in dashed border lines

For each chromosome, in a given disease subgroup, within a defined model, we calculated the mean protein abundance in samples. Protein intensities were then normalized to the respective mean protein value. Normalized protein ratios were aligned based on gene location and visualized. Multiple patients with distinct protein patterns for chromosome X (Fig. 5a and Supplementary Table S5) had structural alterations in chromosome X (Table 1). In particular, patients B-01-Dx and B-08-R2, had trisomy X, patient B-04-Dx had a monosomy X, while patient B-02-Dx had a translocation between chromosome X and 2, t(X;2). Despite a loss of one X chromosome, patient B-04-Dx retained protein features similar to all other patients lacking alterations in chromosome X, irrespective of biological gender category. The correlation scores (Fig. 5a, inset) confirmed these protein changes were stably reflected in matched patient xenografts.

We next validated whether specific genomic aberrations in patients were maintained in their corresponding xenograft leukemia. Based on sample availability, targeted next-generation sequencing (NGS) was performed on a subset of six paired primary and PDX samples using the Oncomine Childhood Cancer Research Assay (OCCRA) [24]. The OCCRA gene panel includes single nucleotide variations (SNVs), copy number variation (CNVs), and gene fusion combinations, that together proved more sensitive for the detection of childhood cancers as against adult-based panels. Mutations detected are detailed in Table 3. An *ETV-RUNX1* fusion in patient B-02-Dx was also identified in the equivalent xenograft leukemia (B-02-Dx-PDXb). While loss of one copy of *SOCS2* and *TET2* was apparent in only B-02-Dx-PDXb. SNVs in ARID1A (Gln563Ter) and in KRAS (Lys117Arg) were retained in B-05-R1 patient and PDX. Additional variations including loss of one copy each of *CDKN2A* and *CDKN2B* occurred in B-05-R1-PDX. For three cases, B-06, B-07, and T-01, the exact alterations were preserved in their respective PDXs. These results support previously known findings that genetic alterations in primary leukemia are mostly retained in PDX models, however, clonal expansion in xenograft leukemia increases the chances for detecting additional tumor suppressor or leukemia driver mutations [7, 27].

**Table 3:** Genomic alterations detected in patient and corresponding PDX by next generation sequencing using the OCCRA gene panel (Lorentzian et al., 2019)

	Disease Type	Sample Origin	OCCRA panel NGS
**1**	B-ALL Dx	**Primary**	
		B-02-Dx	*ETV6-RUNX1* fusion
			SNV: SUZ12 lys690fs, 0.03
		**PDX**	
		B-02-Dx-PDXb	*ETV6-RUNX1* fusion
			CNV: SOCS2 (0.83), TET2 (0.97)
**2**	B-ALL R1	**Primary**	
		B-05-R1	SNV: ARID1A (Gln563Ter, 0.33), KRAS (Lys117Arg, 0.35)
		**PDX**	
		B-05-R1-PDX	SNV: ARID1A Gln563Ter (0.49), KRAS Lys117Arg( 0.52)
			CNV: FASLG (3.67), CDKN2A (0.85), CDKN2B (0.96)
**3**	B-ALL R3	**Primary**	
		B-06-R3	SNV: JAK1 (Val658Phe, 0.48)
			CNV: CDKN2A (0.07), CDKN2B (0.07), PTCH1(0.79), EBF1(1.0)
		**PDX**	
		B-06-R3-PDXb	SNV: JAK1 (Val658Phe, 0.99)
			CNV: CDKN2A (0), CDKN2B (0), PTCH1 (0.76), EBF1 (1.0)
**4**	B-ALL R3	**Primary**	
		B-07-R3	SNV: KRAS Gln61His (0.13)
			CNV: CDKN2A (0.52), RB1 (0.85), P53 (1.37)
		**PDX**	
		B-07-R3-PDXa	SNV: KRAS Gln61His (0.4)
			CNV: CDKN2A (0.33), RB1 (0.22), P53 (1.1)
**5**	T-ALL Dx	**Primary**	
		T-02-Dx	*STIL-TAL1* fusion
			SNV: PTEN (Leu247fs, 0.2)
		**PDX**	
		T-02-Dx-PDX	*STIL-TAL1* fusion
**6**	T-ALL Dx	**Primary**	
		T-01-Dx	CNV: CDKN2A (0.6)
		**PDX**	
		T-01-Dx-PDX	CNV: CDKN2A (0.03)

We then determined if our proteomics data could associate protein patterns to CNVs and SNVs validated by targeted NGS. Deletions in *CDKN2A* on chromosomal region 9 of p21 was detected at a rate of 62% in our sequenced 12-sample cohort (Table 3). Patients verified with a complete loss (CNV = 0) of *CDKN2A*, a well-known tumor suppressor [6, 28], lacked detectable protein amounts (Fig. 5b). Interestingly, patients with one-copy loss of *CDKN2A* had no protein or reduced protein levels in contrast to patients with both copies of the gene (Fig. 5c). Immunoblot tests confirmed our mass spectrometry-based data on CDKN2A (Fig. 5b-d). CDKN2A was only detected in patient primary and xenograft samples with two gene copies.

For patient B-02 with deletions in one copy of *SOCS2* and *TET2* respectively (Table 3), no substantial depletion in SOCS2 and TET2 levels were detected at peptide and protein FDR of 0.01. The same patient had a cryptic *ETV6-RUNX1* (t(12:21)) fusion. Both ETV6 and RUNX1 protein levels did not differ significantly in B-02 in comparison with other diagnostic B-ALL samples.

Reduced protein levels following a hemizygous copy loss in the retinoblastoma protein, RB1, were conserved between patient B-07 and its corresponding PDX, analyzed by targeted NGS. Compared to all sequenced sample pairs, RB1 protein abundance was significantly decreased in B-07 patient and xenograft (Table 3 and Fig. 5e). Additionally, RB1 protein levels were similar in the multiple xenograft samples from patients B-02, B-06, and B-07, which were not analyzed by targeted NGS (Supplementary Fig. S9A).

### PDXs recapitulate targetable gene expression changes in patients

Pediatric cancer drugs target genetic changes in childhood B- and T-cell leukemia that have been linked to patient phenotype [1, 3, 7, 29]. To determine how these gene alterations in pediatric leukemia are presented at the proteome level in patients and in PDXs, we examined the profile of 201 pediatric cancer-related genes and known drug targets [24]. A substantial number of proteins from the OCCRA set of 201 genes were quantified in our sample cohort: 109 based on total protein abundance; 47 phosphoproteins, and N Termini matching to 34 proteins (Fig. 5e, Supplementary Table S6). Spearman’s correlation scores, showed strong similarities between total protein levels in patients and corresponding PDX (Fig. 5f). This was also the case for patients with more than one expanded xenograft mice (Supplementary Fig. S9B). Similarity scores were however lower for T-ALL patients with reduced bone marrow contribution and their corresponding xenografts (T-01-R1 and T-01-R1-PDXa/b, T-03-Dx and T-03-Dx-PDX respectively) (Fig. 5f). The absolute Log_2_ protein levels in patients compared to PDX averaged below 1 in all pediatric ALL subtypes, indicating that the abundance profile of most OCCRA proteins were conserved in PDXs (Fig. 5g). Proteins KMT2A, KMT2D and GNA11 were the most variable proteins. Well-known pediatric cancer-relevant proteins are largely unchanged in primary and xenograft leukemia models. To determine if the observed changes in abundance of the epigenetic regulators KMT2A and KMT2D could lead to a global effect on regulation of their target genes, we retrieved curated lists of protein coding genes regulated by each transcription factor from hTFtarget, a comprehensive repertoire of transcription factor-target relationships for humans [30]. Of the 214 genes regulated by KMT2A, 124 were identified in our dataset, and 102 of these were quantified (Supplementary Table S7). The absolute fold change for quantified proteins between paired patients and PDX is shown in Supplementary Fig. S10A. Proteins ZNF217 and ATXN2 showed the strongest abundance changes between patient and xenograft. KMT2D had 996 curated target genes. We identified 389 and quantified 302 (Supplementary Table S7), with proteins CLUH and RNF138 showing the strongest abundance changes between patient and PDX. (Supplementary Fig. S10B). The total number of *KMT2A* and *KMT2D* targets showing protein abundance change greater than 2-fold in each patient/PDX pair is depicted in Supplementary Fig. 10C. This suggests that differential abundance of KMT2A and KMT2D may directly result in deregulation of several of their target genes.

### PTMs are largely conserved between patient and PDX but proteolysis drives select functional differences not evident at the protein level

To determine if post-translational modification of proteins drives additional functional differences beyond changes in protein abundance we studied the two most abundant and functionally most impactful modifications, protein phosphorylation and proteolytic processing. Here, we compared the average intensity of each feature in patients (excluding non-leukemic patients and patients with low bone marrow involvement) with the corresponding average intensity in PDXs. We found that total protein (r^2^=0.94) and phosphopeptide (r^2^=0.90) intensities correlated better (*p*-value< 0.0001) between the patient and xenograft groups while N termini abundance correlated moderately (r^2^=0.73, p-value< 0.0001) (Supplementary Fig. S11A – F). We next investigated if proteins that are stable between patients and PDX show altered PTMs. For each ALL subtype, proteins that did not significantly change between patient and PDXs (Student’s t-test, q value> 0.05) were evaluated for significant differences (Student’s t-test, q value< 0.05, fold change >=±3) in phosphorylation sites or in neo N termini. No significant changes in phosphosite and phosphopeptide levels that contrasted protein abundance were detected, while a subset of protein N termini indicated differences in proteolytic processing. The unique cleavage pattern of MCM5 in ALL patients and corresponding PDXs further substantiated the existence of proteolytic fingerprints that are indistinguishable at the protein level (Supplementary Fig. S11G).

To evaluate the altered proteolytic processing in detail we characterized protease-generated neo N termini (see Methods for definition) in ALL subtypes using TopFIND [31, 32] and TopFINDER [33]. Neo N termini profiles showed a loss in caspase 1 (CASP1) proteolytic activities in blast cells after transplantation, and a gain in elastase (ELNE) activities in xenografts (Supplementary Fig. S11H and Supplementary Table S8).

**Fig. 6:**
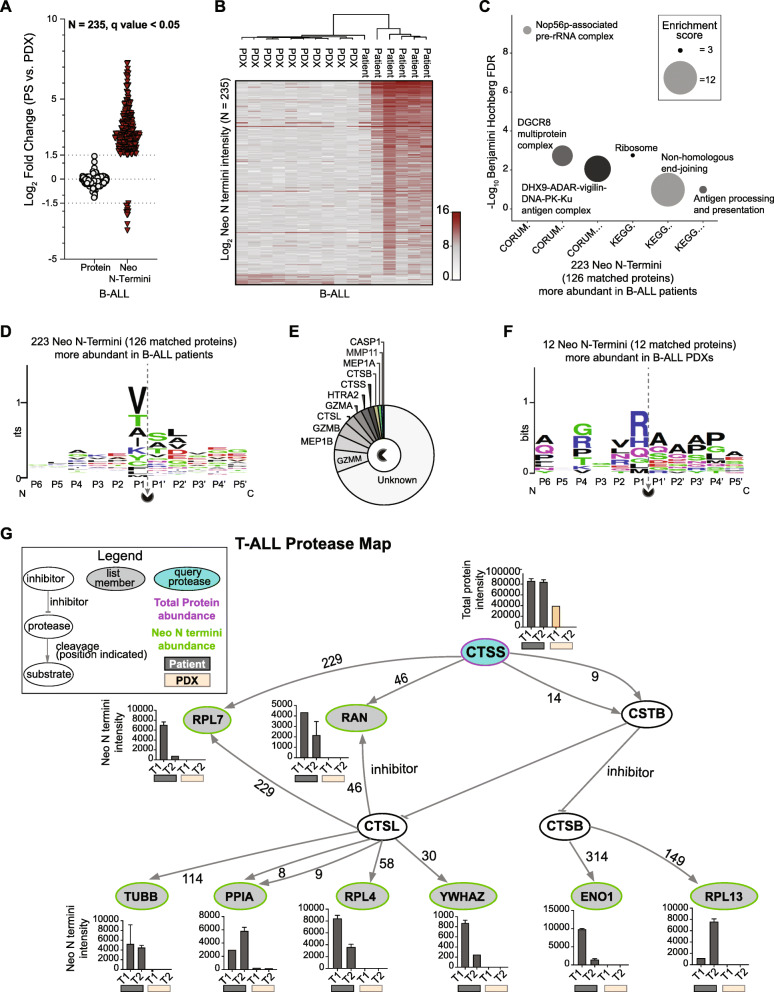
Neo N-terminal PTMs show differences in B-ALL patients and PDXs that are absent at protein level. **a** Proteins (*N* = 235) that remained stable between B-ALL patients and PDXs (Log_2_ fold change < ±1.5) but differed in neo N termini abundance in xenografts and patients (Log_2_ fold change >±1.5, unpaired two-tailed Student’s t-test, FDR < 0.05). **b** Log_2_ abundance plot of 235 differentially regulated neo N termini in patients and corresponding PDXs. **c** Enriched pathway (KEGG) and protein complexes (CORUM) mapped to protein-matched upregulated neo N termini (*N* = 126 proteins from 223 neo N termini). Circle size represents the enrichment score of the category term. **d** Sequence pattern detected for 223 neo N termini enriched in B-ALL patients. **e** Curated proteases with cleavage sites associated with the sequence patterns identified in 223 neo N termini. **f** Sequence pattern detected for 12 neo N termini enriched in B-ALL xenografts. **g** Protease web plot of CTSS interaction network. CTSS (query protease, purple border) mean protein abundance, and Neo N termini mean abundance for list members (proteins with neo N termini in query list, green border) (*N* =198) are plotted in bar graphs. Graphs show intensity of neo N termini quantified in patients (grey bars) and in PDXs (orange bars). Error bars indicate the standard deviation from the mean for replicate DIA-mass spectrometry measurements. The amino acid following the protease N terminal cleavage site (amino acid P1’) is specified for each protease-substrate path

Comparing patients and PDX of B-cell origin (Supplementary Table S9), we identified 235 changing neo N termini in proteins that remained stable (Fig. 6a and b). Specifically, 224 neo termini were higher in patients while 12 were elevated in PDX. To evaluate the functional relevance of these neo termini, biological pathway (KEGG) and protein complex (CORUM) enrichment was performed on proteins from neo N termini unique to patient or PDX. Complexes and pathways enriched in patients were linked to protein pre-processing and maturation, as well as antigen processing and presentation (Fig. 6c). The sequence context for these neo N termini showed P1, P1’ and P2’ as the main specificity conveying positions (Fig. 6d), and they result from cleavage activities of proteases shown in Fig. 6e. Twelve neo N termini downregulated in patients and showed a marked difference in their sequence composition from upregulated termini (Fig. 6f). Their corresponding proteins were enriched in the Histone H3.3 complex (CORUM, Benjamini Hochberg FDR=0.03, enrichment factor = 101.6) and no matching protease cut-site was reported in TopFIND.

In our smaller cohort of two T-ALL patients (blast count> 80%) and corresponding PDXs, 194 neo N termini were dysregulated (189 upregulated, and 9 downregulated) between patients and PDXs (Supplementary Table S10, Supplementary Figs. S12A and B). Enrichment analysis showed similar processes and complexes associated with protein processing and maturation (Supplementary Fig. S12C), comparable sequence motifs and a similar protease map (Supplementary Fig. S12D-F), as was detected for B-ALL patients.

Proteases can cleave other proteases, and protease inhibitors, thereby indirectly regulating the cleavage of substrates of other proteases in vivo [34]. To explore protease interactions, we used PathFINDer [33] to associate protein-level protease changes in leukemic models to direct or indirect cleavage resulting in observed N termini. Cathepsin S (CTSS) abundance was markedly lower or absent in PDX when compared to paired patient samples (Fig. 6g). In addition to the direct CTSS substrates, RPL7 and RAN, neo N termini altered between T-ALL patients and PDX were linked to potential downstream effects of CTSS activity (Fig. 6g). The first nine amino acids of Cystatin B (CSTB) are required for effective inhibition of Cathepsin B (CTSB) and Cathepsin L (CTSL) [35]. Cleavage of Cystatin B by elevated Cathepsin S at position 9 could lead to increased activity of CTSB and CTSBL, and subsequent increase in neo termini of their substrates. Known products of CTSB and CTSL processing were present in patients and absent or reduced in xenografts, suggesting an activity-linked cleavage pattern for Cathepsin S in T-ALL patients. Proteins directly or indirectly linked to Cathepsin S in vivo proteolytic activity had functional roles in apoptotic signaling (ENO1, YWHAZ), RNA metabolism (RAN, RPL7, RPL4, RPL13, YWHAZ) and protein folding (PPIA). A similar protease network was associated with neo N termini alterations in B-ALL (Supplementary Fig. S13A).

Although the vast majority of proteolytic processing events did not significantly differ between patient and PDX, a subset of the N terminome was significantly altered in PDX. The phosphorylation sites covered in this study did not significantly change with the transition from the patient to the mouse xenograft.

## Discussion

Patient-derived xenografts provide a vital means to simulate human diseases, understand disease biology, and most importantly, to develop therapeutic approaches. It was recently shown [36, 37] (and us, in review) that diagnosis-PDX recapitulate the population complexity of the primary samples, with some expansion of minor subpopulations. Serial passaging could therefore result in significant changes in population dominance as a result of clonal selection, which would then lead to differences between multiple expansions and the primary cancer as a result of loss of cell heterogeneity. Comparative studies on patient and PDXs with primary engraftments of patient leukemia have established that genetic and epigenetic abnormalities associated with pediatric B- and T-cell leukemias are recapitulated in PDX models [7–10, 27]. The functional relevance of proteins set them apart as valuable indicators of disease phenotypes, and as therapeutic targets. It is therefore important, and of due time, to determine how accurately protein functional abnormalities in pediatric leukemia patients are reflected in PDX models. Such knowledge is of particular importance to determine if PDX models can be used to study response to treatments targeting signaling pathways in preclinical studies and if a particular model is suitable to study select aspects of cancer biology. While our ALL clinical and PDX cohort is insufficient for robust discovery of candidate disease markers, it offers for the first time, a global molecular view of disease-driven changes in the proteome of pediatric ALL subtypes, and evaluates how this is recapitulated in the commonly used pediatric ALL xenograft NSG mouse model. PDXs capably propagated leukemias from patients with minimal bone marrow contribution, and more importantly retained a leukemia protein landscape similar to other xenografts.

This study provides initial insights into the fate of ALL-related PTMs in primary xenografts. Post-translational protein modification by phosphorylation and proteolysis are amongst multiple modification events that expand the functional roles of a protein. Proteolytic processing assessed by N terminome profiling, and phosphorylation profiles of patients are clearly distinct from non-leukemic bone marrow mononuclear cells and show moderate correlation with PDXs. This data shows that disease-related, steady-state proteome profiles are maintained in PDX and patient host. Neo N termini patterns from limited proteolysis act as fingerprints to identify active proteases and their corresponding cleavage sites in different cellular conditions and locations [18]. Indeed, these patterns revealed non-normal enriched proteolytic activities in pediatric ALL cell lines, patients and PDXs, some of which were undetectable at the protein level. These discrepancies in proteome representation should be considered when PDX models are utilized in preclinical investigation. This data therefore provides a repertoire of N termini that could be further explored for biological and potential therapeutic relevance in pediatric ALL.

Childhood leukemias have diverse genomic aberrations [1, 3, 29], known to be replicated in xenografts [7, 38]. Pediatric B-ALL cases are often associated with recurrent chromosome translocations [39]. With proteomics, we confirmed that structural defects and mutations in patients are faithfully recapitulated in their xenografts. An overview of the 219 protein abundance features associated to 883 protein-coding genes on chromosome X indicated that patients with trisomy X had an abnormal protein profile, as did their paired PDXs (Fig. 5a). Of note, the protein profile of 84% of pediatric cancer-related genes validated from extensive genomic and transcriptomic characterizations [3, 7, 24, 29, 40] was essentially preserved in xenograft cells after expansion in mice. The protein level of KMT2A and KMT2D epigenetic regulators, and a few of their targets were found to fluctuate consistently between patients and their matched PDX leukemia. This could indicate that discrepancies in the regulation of KMT2A and KMT2D in engrafted leukemia also affect specific target genes. However, additional studies would be vital to confirm this.

Other findings in this study exposed deficient or missing components in the PDX model that may compromise its capacity to replicate specific processes and signaling pathways in patients. Genomic and epigenomic characterization of T-cell leukemia PDX models did show reduced immune/defense responses and limited stimulation of cytokine response in xenografts compared to their paired primary leukemias [7, 27]. Our study confirmed these findings and further showed that such compromised immune and defense responses are evident in the proteome of B- and T-ALL xenografts.

Our data profiled the cellular levels of 180 proteins linked to cytokine-mediated signalling pathways, cytokine production, and cytokine metabolic processes. While the majority of these proteins were stable between PDX and patients, five proteins critical for cytokine signalling were markedly deficient or missing in B- and T- cell xenograft leukemias, as well as in 380 cell line-derived xenograft (Supplementary Fig. S13). Proteins S100A8, S100A9, S100A12 are important for cytokine production, while the secreted glycoprotein, CHI3L is a Th2 promoting cytokine. Our data also identified increased abundance of proteins linked to cell cycle and mitosis in PDXs over patients, and in CDXs over cell lines, suggesting increased proliferation in xenografted leukemia cells. This is in line with genome-wide DNA analysis on paired T-ALL patients and PDXs which suggested that increased proliferation contributes to the selective advantage of engrafted cells [7]. Increase in cell cycle and proliferation markers may also be explained by a lack of spatial constraints for xenografted cells localized to the spleen or liver (in CDXs) relative to patient cells restricted by the bone marrow confinement. Lastly, with different associated micro-environments, cultured cell lines and mice consume and generate energy through distinct pathways. It is therefore not surprising that proteins involved in the generation of precursor metabolites differ strikingly in both systems (Fig. 4i).

This study provides important findings on the stability of protein molecules in pediatric ALL after xenotransplantation in NSG mice. Since the study was limited to one specimen per patient disease condition, we could not conclusively determine if xenografts are adequate to model patient-specific differences mediated by proteolysis and/or phosphorylation, and if these specific differences include treatment relevant pathways. This would warrant targeted perturbation with compounds of interest as well as comprehensive longitudinal proteome profiling which is challenging as repeat biopsy collection from young patients is ethically problematic. The study does however provide strong support for xenografts as accurate model of pediatric acute leukemias, and offers valuable data on the proteome of pediatric ALL blast cells. Furthermore, our rationale to include patient samples with two xenografts provided first insights into proteome similarities in PDXs derived from the same patient origins. Also, given the limited samples analyzed, our phosphorylation experiment is limited in the capture of tyrosine kinases, which are known to be highly dysregulated in pediatric ALL. Additional studies combining tyrosine kinase enrichment with our established protocol would reveal the extent to which disease-linked phosphorylation patterns are maintained in PDXs.

## Conclusion

In summary, our study shows that pediatric leukemia cells principally maintain their protein abundance pattern when xenografted into immunocompromised NSG mice. Total protein and protein modification landscapes appear differently affected by the host, leukemia type and patient of origin. Phosphorylation and proteolysis are largely correlated between patients and xenografts but less robustly recapitulate patient and xenograft pairing. These differences underscore the need to characterize not only protein abundance but also key post-translational modifications in model organisms. Importantly, our study showed that PDX models, even if they originate from an unrelated patient, far better reflect a patients leukemia than established cell lines. Overall, PDX models are a well-suited proxy for investigation of subtype and patient-specific disease biology, and clinical evaluation of new therapeutic approaches.

## Supplementary Information

**Additional file 1.**

**Additional file 2.**

**Additional file 3.**

**Additional file 4.**

**Additional file 5.**

**Additional file 6.**

**Additional file 7.**

**Additional file 8.**

**Additional file 9.**

**Additional file 10.**

**Additional file 11.**

**Additional file 12.**

**Additional file 13.**

**Additional file 14.**

**Additional file 15.**

**Additional file 16.**

**Additional file 17.**

**Additional file 18.**

## Data Availability

The datasets generated and analyzed in this study are available in the Proteomics Identification Database (PRIDE) with the provided accession numbers: PXD016545 for HUNTER N terminome enrichment, PXD016547 for phosphoprotein enrichment, and PXD016548 for global protein study on B-ALL and T-ALL cell lines, patient samples and PDXs. Datasets for global protein analysis on 380 cell lines and 380 cell line-derived xenograft can be accessed with accession number PXD023697.

## Abbreviations

ALL: Acute Lymphocytic Leukemia
B-ALL: B-cell Acute Lymphocytic Leukemia
T-ALL: T-cell Acute Lymphocytic Leukemia
T-LBL: T-cell Acute Lymphoblastic Leukemia
PTM: Post-translational Modification
PDX: Patient-derived Xenograft
OCCRA: Oncomine Childhood Cancer Research Assay
SNV: Single Nucleotide Variation
CNV: Copy Number Variation
HUNTER: High-efficiency Undecanal-based N Termini Enrichment
LC-MSMS: Liquid Chromatography Tandem Mass Spectrometry
DDA: Data Dependent Acquisition
DIA: Data Independent Acquisition
